# Supplementary material for: Mapping the evolution of fertility support policies in China: A content and instrumental analysis
Source: PLoS One. 2025 Oct 9;20(10):e0332137. doi: 10.1371/journal.pone.0332137 (PMC12510515; doi:10.1371/journal.pone.0332137)
Supplement: S1 Appendix — (ZIP) [file pone.0332137.s001.zip › S1 Appendix. 226 original policy documents/22-国家卫生计生委关于贯彻落实中共中央、国务院关于实施全面两孩政策改革完善计划生育服务管理的决定的通知(FBM-CLI-4-262156).doc]

国家卫生计生委关于贯彻落实中共中央、国务院关于实施全面两孩政策改革完善计划生育服务管理的决定的通知

**发布部门：** [国家卫生和计划生育委员会(已撤销)](https://www.pkulaw.com/law/chl?Aggs.IssueDepartment=60319) [机构沿革](javascript:void(0))

**发文字号：**国卫指导发〔2016〕1号

**发布日期：**2016.01.05

**实施日期：**2016.01.05

**时效性：** [现行有效](https://www.pkulaw.com/law/chl?Aggs.TimelinessDic=01)

**效力级别：** [部门规范性文件](https://www.pkulaw.com/law/chl?Aggs.EffectivenessDic=XE0302)

**法规类别：** [计划生育管理](https://www.pkulaw.com/law/chl?Aggs.Category=09705)

国家卫生计生委关于贯彻落实中共中央、国务院关于实施全面两孩政策改革完善计划生育服务管理的决定的通知
（国卫指导发〔2016〕1号）

各省、自治区、直辖市卫生计生委，新疆生产建设兵团卫生局、人口计生委，解放军、武警部队计划生育领导小组办公室，中直机关、中央国家机关人口计生委，委机关各司局，委直属和联系单位：
　　为贯彻落实《中共中央 [国务院关于实施全面两孩政策改革完善计划生育服务管理的决定](https://www.pkulaw.com/chl/8564756bae805261bdfb.html)》（中发〔2015〕40号，以下简称《决定》），全面做好新形势下的计划生育工作，现就有关事项通知如下：
　　**一、**统一思想，深入学习领会《决定》精神实质
　　《决定》充分肯定了计划生育工作取得的巨大成就。长期以来，我国坚持计划生育基本国策，实施人口与发展综合决策，不断完善计划生育政策，走出了一条中国特色统筹解决人口问题的道路。人口过快增长的势头得到有效控制，资源、环境压力有效缓解，妇女儿童发展状况极大改善，人口素质明显提高，促进了经济快速发展和社会进步，有力支撑了改革开放和社会主义现代化事业，为全面建成小康社会奠定了坚实基础。我国实行计划生育也为世界人口发展和减贫作出了重大贡献，树立了负责任大国的良好形象。
　　《决定》指出，当前我国正处于人口大国向人力资本强国转变的关键时期。必须从全局和战略高度出发，充分认识坚持计划生育基本国策的重要性和长期性，立足国情，遵循规律，正确处理当前与长远、总量与结构、人口与资源环境的关系，逐步调整完善生育政策，促进人口长期均衡发展，最大限度发挥人口对经济社会发展的能动作用，牢牢把握战略主动权。
　　《决定》认为，实施全面两孩政策、改革完善计划生育服务管理，是促进人口长期均衡发展的重大举措，有利于优化人口结构，增加劳动力供给，减缓人口老龄化压力；有利于促进经济持续健康发展，实现全面建成小康社会的奋斗目标；有利于更好地落实计划生育基本国策，促进家庭幸福与社会和谐。《决定》明确要求，各级党委政府要增强责任感和使命感，用法治的思维、创新的精神和务实的作风，不断探索新形势下落实计划生育基本国策的体制机制和方式方法，使计划生育成为惠及亿万家庭的甜蜜事业。
　　《决定》明确了新时期计划生育工作的指导思想、基本原则和主要目标。要高举中国特色社会主义伟大旗帜，全面贯彻党的十八大和十八届二中、三中、四中、五中全会精神，以邓小平理论、“三个代表”重要思想、科学发展观为指导，深入贯彻习近平总书记系列重要讲话精神，坚持“四个全面”战略布局，以增进家庭和谐幸福、促进人口长期均衡发展为主线，坚持计划生育基本国策，按照“以人为本、创新发展、法治引领、统筹推进”的原则，统筹推进生育政策、服务管理制度、家庭发展支持体系和治理机制综合改革，努力实现规模适度、素质较高、结构优化、分布合理的人口均衡发展，促进人口与经济社会、资源环境协调可持续发展，为推进社会主义现代化建设、实现中华民族伟大复兴的中国梦提供坚实基础和持久动力。

　　**二、**把握重点，贯彻落实《决定》提出的各项任务
　　（一）稳妥扎实有序实施全面两孩政策。各省（区、市）要全面分析本地人口形势，做好群众生育意愿调查，科学测算全面两孩政策的目标人群，综合分析全面两孩政策对经济、社会以及基本公共服务等方面的影响，深入评估风险和可能出现的问题，认真研究制定全面两孩政策实施方案。实施方案、人口变动预测、配套政策措施、风险防控预案以及修订地方人口与计划生育条例的主要建议等相关材料，由省级政府及时报我委备案。实施方案提出的目标任务和工作措施将作为工作督查和评估的重要参考。
　　要根据《决定》精神和新修订的《[人口与计划生育法](https://www.pkulaw.com/chl/18c6c388fc14eaa9bdfb.html)》等法律、行政法规规定，总结计划生育服务管理的成熟经验，及时提请省级人大或其常委会修订本省（区、市）人口与计划生育条例。重点对生育调节、避孕节育服务、生育过程管理、奖励与社会保障、出生人口性别比治理、社会抚养费征收管理、法律责任等相关条款进行修订。遵循法制统一原则，注意与其他省（区、市）的政策协调，做好全面两孩政策实施前后奖励扶助和社会制约政策的衔接，维护公民合法权益。不再实施鼓励公民晚婚晚育、一对夫妻生育一个孩子的奖励政策，构建支持按政策生育的服务保障体系。综合考虑公平统一和保持政策连续性等原则，合理确定再婚等特殊情形再生育政策。实行生育登记服务制度，优化再生育办理流程，推行网上办理、一站式服务和承诺制，为群众提供高效便捷服务。
　　（二）大力提升妇幼保健和计划生育服务管理水平。以“调整存量、做优增量、补齐短板、提升能力”为原则，力争在“十三五”前期解决妇幼健康服务资源总体不足和结构性短缺的供需矛盾。将妇幼保健和计划生育服务机构建设作为重点，完善设施设备，改善服务条件。提高孕产妇、新生儿危急重症救治能力，健全会诊、转诊网络，保障母婴安全。开展免费孕前优生健康检查，加强高龄孕产妇、再生育和不孕不育人群的服务和指导，注重发挥中医药的特色与优势。为符合条件准备再生育人群免费提供取环、复通等计划生育基本技术服务。科学规划、统筹做好出生缺陷综合防治，提高出生人口素质。全面落实避孕方法知情选择，对已生育两个子女的夫妻提倡使用长效避孕措施，提高避孕药具服务可及性，减少非意愿妊娠。结合实际，组织开展计划生育生殖健康科普宣传和服务，提高群众生殖健康水平。
　　认真做好县、乡级卫生计生机构改革，促进卫生计生工作融合，稳定和加强基层计划生育工作网络和队伍，充实县级卫生计生部门计划生育工作力量，健全乡镇（街道）计生办，配强配齐村居（社区）计生专干，妥善解决好村级计生专干的报酬待遇、养老保障等问题。切实加强县、乡级计划生育协会的组织和能力建设，充分发挥计划生育协会的生力军作用。深入开展新一轮计划生育优质服务先进单位创建活动，深化流动人口全国“一盘棋”机制建设，综合治理出生人口性别比偏高问题。严禁将落实计划生育政策与落户、入托、入学等挂钩，建立各相关部门执法配合、政策衔接、信息共享的计划生育综合治理机制。严格控制政策外多孩生育，依法征收社会抚养费。
　　（三）构建有利于计划生育的家庭发展支持体系。加大对计划生育家庭扶助力度，切实保障计划生育家庭合法权益。关怀计划生育特殊家庭，妥善解决他们在生活照料、养老保障、大病治疗、精神慰藉等方面的困难和问题。按照“老人老办法，新人新办法”的原则，完善并落实计划生育家庭奖励扶助制度、特别扶助制度、“少生快富”工程以及其他各项奖励扶助政策；全面两孩政策实施前，已经享受独生子女父母奖励政策的，按规定的条件、标准、年限继续执行；全面两孩政策实施后，没有再生育的计划生育家庭，符合条件的按规定纳入计划生育家庭奖励扶助制度和特别扶助制度；只生育一个孩子的，不再享受独生子女父母奖励政策；独生子女家庭再生育的，停止享受独生子女父母奖励政策，此前已享受的不再退还。
　　按照《决定》要求，建立完善包括生育支持、幼儿养育、青少年发展、性别平等、老人赡养、病残照料等在内的家庭发展政策和服务体系。完善计划生育奖励假制度，参照本地原人口与计划生育条例中有关公民晚育假期奖励的规定，制定对符合政策生育夫妇延长生育假奖励等相关政策措施，保障女性就业、休假等合法权益。

　　**三、**求真务实，把学习贯彻《决定》不断引向深入
　　（一）强化组织领导。各省（区、市）卫生计生委要将贯彻落实《决定》作为卫生计生工作的重中之重，作为推进健康中国建设的重要任务，坚持一把手亲自抓、负总责，分管领导具体抓，明确相关处室的职责，狠抓各项任务的落实。坚持和完善计划生育目标管理责任制，合理设置考核项目和权重，改革考核评估方法，细化“一票否决”的范围，严格兑现奖惩。做好信访维稳工作，确保政策平稳实施。国家卫生计生委机关各司局要结合职能职责，加大对工作的指导力度。
　　（二）强化宣传倡导。要制定宣传方案，编写宣传提纲和宣传读本，深入宣传计划生育工作的伟大成就，大力宣传坚持计划生育基本国策、调整完善生育政策的重要意义，弘扬主旋律，积累正能量。策划、制作一批总结计划生育历程、反映人口形势、揭示人口发展规律、宣传基层先进典型等有特色的影视宣传品，组织制作通俗易懂的融政策、知识、服务信息为一体的宣传折页，因地制宜地开展进村入户的宣传工作，营造良好舆论氛围。做好政策解读和舆情监测，充分利用互联网、广播、电视、报刊等渠道，积极开展宣传工作，及时回应社会关切，防止恶意炒作。深入开展第五阶段婚育新风进万家活动。
　　（三）强化出生监测。健全县乡村三级出生监测网络，推进生育登记、孕产期保健、住院分娩、出生医学证明、儿童预防接种等信息共享，实现国家与省级计划生育信息互联互通；加强与公安、民政、人力资源社会保障、教育等部门协作，建立健全人口基础信息特别是流动人口动态信息共享机制，准确掌握新生儿入户、婚姻登记、儿童参加社会保障、入学等情况，及时把握出生人口动态。
　　各省（区、市）卫生计生委要将贯彻落实《决定》的情况向我委报告，我委将适时组织督查。

国家卫生计生委
2016年1月5日

© 北大法宝：（[www.pkulaw.com](https://www.pkulaw.com/)) 专业提供法律信息、法学知识和法律软件领域各类解决方案。北大法宝为您提供丰富的参考资料，正式引用法规条文时请与标准文本核对。
欢迎查看所有[产品和服务](https://www.pkulaw.com/helps/2.html)。法宝快讯：[如何快速找到您需要的检索结果？](https://www.pkulaw.com/helps/70.html)[法宝](https://www.pkulaw.com/helps/69.html)V6有何新特色？

扫描二维码阅读原文

**原文链接：**<https://www.pkulaw.com/chl/2f46ebef581e3821bdfb.html>
